# Autonomic Changes in Juvenile-Onset Huntington’s Disease

**DOI:** 10.3390/brainsci10090589

**Published:** 2020-08-26

**Authors:** Jordan L. Schultz, Peg C. Nopoulos

**Affiliations:** 1 Department of Psychiatry, Carver College of Medicine at the University of Iowa, Iowa City, IA 52242, USA; peggy-nopoulos@uiowa.edu; 2 Department of Neurology, Carver College of Medicine at the University of Iowa, Iowa City, IA 52242, USA; 3 Department of Pediatrics, Carver College of Medicine at the University of Iowa, Iowa City, IA 52242, USA

**Keywords:** juvenile-onset Huntington’s Disease, autonomic, neurodegeneration

## Abstract

Patients with adult-onset Huntington’s Disease (AOHD) have been found to have dysfunction of the autonomic nervous system that is thought to be secondary to neurodegeneration causing dysfunction of the brain–heart axis. However, this relationship has not been investigated in patients with juvenile-onset HD (JOHD). The aim of this study was to compare simple physiologic measures between patients with JOHD (*n* = 27 participants with 64 visits) and participants without the gene expansion that causes HD (GNE group; *n* = 259 participants with 395 visits). Using data from the Kids-JOHD study, we compared mean resting heart rate (rHR), systolic blood pressure (SBP), and diastolic blood pressure (DBP) between the JOHD and GNE groups. We also divided the JOHD group into those with childhood-onset JOHD (motor diagnosis received before the age of 13, [*n* = 16]) and those with adolescent-onset JOHD (motor diagnosis received at or after the age of 13 [*n* = 11]). We used linear mixed-effects models to compare the group means while controlling for age, sex, and parental socioeconomic status and including a random effect per participant and family. For the primary analysis, we found that the JOHD group had significant increases in their rHR compared to the GNE group. Conversely, the JOHD group had significantly lower SBP compared to the GNE group. The JOHD group also had lower DBP compared to the GNE group, but the results did not reach significance. SBP and DBP decreased as disease duration of JOHD increased, but rHR did not continue to increase. Resting heart rate is more sensitive to changes in autonomic function as compared to SBP. Therefore, these results seem to indicate that early neurodegenerative changes of the central autonomic network likely lead to an increase in rHR while later progression of JOHD leads to changes in blood pressure. We hypothesize that these later changes in blood pressure are secondary to neurodegeneration in brainstem regions such as the medulla.

## 1. Introduction

Huntington’s Disease (HD) is an inherited, neurodegenerative disease that causes motor, cognitive, and behavioral symptoms [1]. These symptoms are thought to be caused by striatal degeneration, which is the hallmark of HD [2,3]. However, there are myriad symptoms that can also occur as a result of the neurodegenerative processes associated with HD. Dysfunction of the autonomic nervous system (ANS) has been described in patients with adult-onset HD (AOHD) [4,5,6,7,8]. Specifically, patients with AOHD seem to have enhanced sympathetic tone compared to healthy controls. Structural and functional changes in the central autonomic network (CAN) of the brain are thought to drive autonomic dysfunction in HD [9,10]. Patients with juvenile-onset HD (JOHD) represent a rare group of individuals with significant neurodegeneration that begins very early in life, but physiologic measures of ANS function have never been reported in this patient population. Given the unique neurodegenerative changes that occur in JOHD, we would hypothesize that these patients would demonstrate some signs of a dysregulated ANS that occurs secondary to brain atrophy. We leveraged a large dataset of patients with JOHD to test the hypothesis that patients with JOHD have a dysregulated ANS with a propensity for enhanced sympathetic tone compared to a control group. This study has the potential to advance our understanding how dysfunction in the CAN may impact peripheral measures of ANS function in JOHD.

## 2. Materials and Methods

### 2.1. Participants

For these analyses, we utilized data from the Kids-HD and Kids-JOHD studies [11,12,13,14]. Both studies were longitudinal neuroimaging studies that ran in parallel. They recruited participants from around the country who were between the ages of 6 and 26 years old to the University of Iowa. The Kids-HD study recruited participants with a family history of HD (parent or grandparent with a known history of HD). These participants underwent genetic testing to determine whether they were gene carriers of the mutation that causes HD. The results of this genetic testing were not revealed to the participants, their family members, or the research staff. One research team member received the genetic results and anonymized them. This allowed for the ethical conduct of genetic testing in children. Additionally, the Kids-HD study recruited a cohort of healthy controls without a family history of HD. For the present analysis, all participants from the Kids-HD study with a CAG repeat length of less than 36 were included in the GNE group. The Kids-JOHD study, in comparison, recruited participants with a known diagnosis of JOHD based on molecular confirmation and a diagnosis provided by a neurologist. Participants with a CAG repeat length of 36 or above who were not symptomatic were excluded from the current analysis.

The JOHD group was first evaluated as a whole. We then split the group into those with childhood-onset JOHD and those with adolescent-onset JOHD. Childhood-onset JOHD is defined as an age of motor diagnosis that occurred before the age of 13, while adolescent-onset JOHD occurred between the ages of 13 and 21.

There were 27 participants in the JOHD group accounting for 64 visits, and there were 259 participants in the GNE group accounting for 395 visits. Among the JOHD group, three participants had five visits, two participants had four visits, five participants had three visits, nine participants had two visits, and eight participants only had one visit. In the GNE group, 12 participants had four visits, 21 participants had three visits, 58 participants had two visits, and 168 participants only had one visit. Among the JOHD group, 11 of the 27 participants were classified as having adolescent-onset JOHD and the other 16 were classified as having childhood-onset JOHD.

### 2.2. Statistical Analyses

For the primary outcome of interest, the physiologic measures of resting heart rate (rHR), systolic blood pressure (SBP), and diastolic blood pressure (DBP) were analyzed. These measures were collected in the Clinical Research Unit at the University of Iowa by trained professionals with equipment that is well maintained and calibrated regularly. We constructed linear mixed-effects regression models to compare the estimated mean differences in these measures between the GNE group and the JOHD group controlling for age, sex, the use of medications that may increase blood pressure, the use of medications that may decrease blood pressure and parental socioeconomic status as well as random effects per participant and per family to account for similarities among siblings. Because we were investigating children and young adults, the use of medications, such as stimulants, that may impact blood pressure were controlled for. Medications that were considered to increase blood pressure were stimulants, including amphetamine and methylphenidate-based products. Medications that were considered to lower blood pressure were carbidopa-levodopa, clonidine, guanfacine, and any anti-hypertensive medications. These models were run to first evaluate the JOHD group together, and then again to compare the childhood-onset JOHD and adolescent-onset JOHD groups to the GNE groups. We performed two unplanned sensitivity analyses. First, we repeated the primary analyses that compared rHR, SBP, and DBP between the JOHD participants and the control group after removing all participants visits from participants who were using medications that increased or decreased blood pressure. Next, we evaluated the relationship between duration of disease and rHR, SBP, and DBP among participants in the JOHD group only using linear mixed-effects regression analyses. Since these models only included participants with JOHD, we added CAG repeat length as a covariate in the models. The models were also adjusted for age, the use of medications that increase blood pressure, and the use of medications known to decrease blood pressure.

RStudio was used for all statistical analyses and a *p*-value of <0.05 was considered statistically significant.

## 3. Results

### Primary Outcomes

Baseline demographics between the groups are outlined in Table 1.

Participants in the JOHD group had significant elevations in their rHR compared to the GNE group. However, the mean SBP in the JOHD group was significantly decreased in the patients with JOHD compared to the GNE group. The mean DBP was lower in the JOHD group as well, but the results did not reach statistical significance (Table 2).

**Figure 1:**
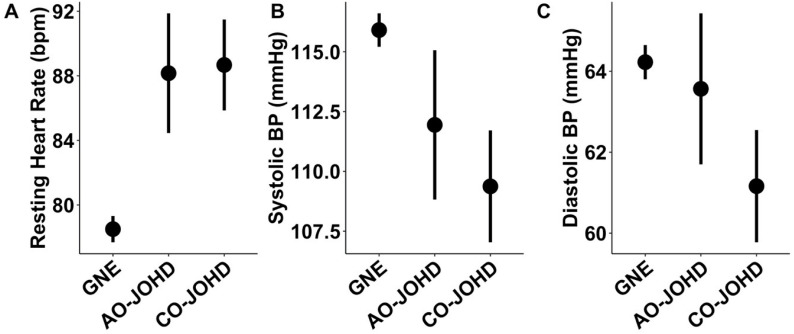
(**a**) Resting heart rate was significantly elevated in the adolescent-onset and childhood-onset JOHD groups compared to the GNE group. (**b**) Systolic BP was significantly decreased in the childhood-onset JOHD group compared to the GNE group. There was a gradual decrease in SBP from the GNE group to the adolescent-onset and childhood-onset JOHD groups. (**c**) Diastolic BP was significantly decreased in the childhood onset JOHD group compared to the GNE group. AO-JOHD: adolescent-onset JOHD; BP: blood pressure; Bpm: beats per minute; CO-JOHD: childhood-onset JOHD; GNE: gene-non-expanded group.

The mean rHR of participants with adolescent-onset JOHD (87.79 ± 3.72 bpm) was significantly elevated compared to the GNE group (78.50 ± 0.83 bpm; *t* = 2.44, *p* = 0.016). The childhood-onset JOHD group had a mean rHR of 89.12 ± 3.11 bpm, which was also significantly elevated relative to the GNE group (*t* = 3.27, *p* = 0.001). The childhood-onset and adolescent-onset JOHD groups did not differ significantly from one another (*p* = 0.786) (Figure 1a). The mean SBP of participants with adolescent-onset JOHD was 111.79 ± 3.14 mmHg compared to 116.00 ± 0.71 in the GNE group (*t* = −1.30, *p* = 0.195). There was a significant difference between the childhood-onset JOHD group and the GNE groups, though. Specifically, the childhood-onset group’s mean SBP was 108.66 ± 2.63 mmHg (*t* = −2.66, *p* = 0.0084). The difference between the childhood-onset and adolescent-onset groups was not statistically significant (*p* = 0.451) (Figure 1b). The mean DBP of the GNE group was 64.26 ± 0.43 mmHg. The adolescent-onset group’s mean DBP was lower (63.11 ± 1.84 mmHg) compared to the GNE group, but not significantly so (*t* = ‒0.609, *p* = 0.543). However, the childhood-onset JOHD group’s mean DBP was significantly lower (60.99 ± 1.55 mmHg) compared to the GNE group (*t* = −2.01, *p* = 0.045) (Figure 1c).

## 4. Discussion

Patients with HD produce a mutant form of the huntingtin protein. This protein is widely expressed in the brain, but also in peripheral tissues [15]. Consequently, peripheral manifestations of HD have been hypothesized to be caused by cellular dysfunction caused by expression of the mutant huntingtin protein in those tissues, independent of the known neurodegenerative process of HD. However, expression of the huntingtin protein is undetectable in the heart [15] and mouse models of HD have revealed changes to the cardiovascular system in the absence of mutant huntingtin aggregates in cardiac tissues, even at end stages of the disease [9]. Therefore, measured changes of physiologic markers of ANS function are more likely to be a secondary marker of neurodegeneration affecting central pathways in areas such as the CAN. Here, we have demonstrated for the first time that patients with JOHD have significant changes in physiologic measures of ANS function compared to healthy controls. Importantly, we hypothesize that these changes are a direct consequence of pathologic changes that occur in the central nervous system of patients with JOHD rather than peripheral manifestations of the disease.

**Figure 2:**
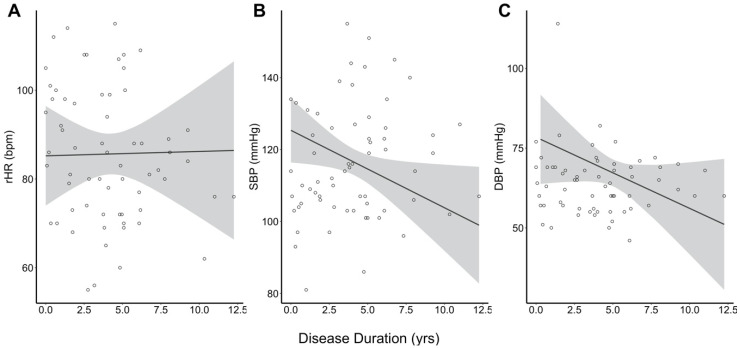
(**A**) Disease duration among patients with JOHD did not significantly predict rHR. (**B**) Systolic BP and (**C**) diastolic BP become significantly more decreased as disease duration increased in the JOHD group. Bpm: beats per minute; DBP: diastolic blood pressure; rHR: resting heart rate; SBP: systolic blood pressure.

Similar to previous reports of enhanced sympathetic tone in patients with AOHD, patients with JOHD had elevations in their rHR compared to healthy controls. However, the patients with JOHD had decreases in their SBP and DBP relative to the control group, which was unexpected. It is unclear what is driving the observed decrease in blood pressure in JOHD, but we hypothesize that earlier neurodegenerative changes may affect regions within the CAN that impact rHR. As patients progress through their disease, we believe that brain regions that are more closely related to blood pressure control are impacted. For example, the medulla oblongata receives afferent signals from the baroreceptors and affects blood pressure control [16]. The medulla has not been widely described as a primary area of neurodegeneration in HD using neuroimaging techniques. However, a post-mortem analysis demonstrated degeneration in areas of the brainstem in patients with HD [17]. This seems to fit with our hypothesis because neuroimaging studies are typically conducted in patients who have not reached the end stages of the disease, but post-mortem analyses obviously would mostly represent patients with end-stage disease, similar to patients with JOHD. To further investigate this theory, we conducted an unplanned analysis to investigate the relationship between disease duration and physiologic measures of cardiac function in the JOHD group. We found no significant relationship between disease duration and rHR (*p* = 0.936; Figure 2a). This supports the notion that rHR becomes significantly elevated early in the disease course of JOHD but does not seem to continue to worsen as the disease progresses. However, there were significant, negative correlations between disease duration and SBP (*t* = −2.20, *p* = 0.037; Figure 2b) and DBP (*t* = −2.13, *p* = 0.044; Figure 2c). These results further confirm our hypothesis that rHR is impacted earlier in the disease process of JOHD and changes in BP may be indicative of later-stage neurodegeneration.

This analysis is the first report (to the best of our knowledge) of physiologic markers of cardiac function being disrupted in JOHD. These results come from one of the largest datasets of patients with JOHD in the world. Despite this, there are important limitations to our work. As noted above, these results only allow us to report associations but are not meant to demonstrate a causative relationship between rHR and SBP in JOHD. Similarly, the Kids-JOHD and Kids-HD studies were not focused on cardiovascular measures. While all participants had their vital signs collected in a similar setting with similar equipment by trained medical professionals, confounding factors related to the collection of rHR and SBP could have been present and may have affected these results. Additionally, we recognize that rHR and SBP are surrogate measures of ANS function. Therefore, future studies focused on investigating ANS function in JOHD should collect more precise measures, such as heart rate variability or baroreflex sensitivity. Another limitation is the potential influence of medication use on these results. We have attempted to control for this confounder by including the use of medication that increase or decrease blood pressure as a covariate in all models. However, this may not have adequately controlled for the impact of medications. To further ensure that medication use was not significantly influencing these results, we performed an unplanned sensitivity analysis where we repeated the primary analyses in participants who were not taking a medication that is known to impact blood pressure. After doing this, the participants in the JOHD group still had a significantly elevated rHR and a significantly lower SBP compared to the GNE group. Interestingly, the JOHD group also had a significantly lower DBP compared to the GNe group in this analysis. While medication use is still an important confounder, this sensitivity analysis seems to indicate that the use of these medications is not significantly impacting the reported results. It is important to note that we hypothesize that the measured changes in rHR, SBP, and DBP in patients with JOHD is mediated by CNS alterations given that JOHD is a neurodegenerative disease. However, further research is needed to support this hypothesis. It is possible that the observed changes are mediated by the peripheral nervous system, the cardiovascular system (including physical fitness), and metabolic rate. Further studies are required to determine the root cause of autonomic dysfunction in JOHD. Lastly, it is important to note that the lower blood pressures in the JOHD group were at rest. Previous reports in patients with Parkinson’s Disease and Multiple System Atrophy performed orthostatic tests to determine whether patients had a precipitous drop in their BP when going from lying down to standing up, which is more indicative of autonomic function. The present study did not perform any specific measures that were meant to perturb the autonomic nervous system. Therefore, the patterns identified at rest are only theorized to be associated with central changes that could affect the cardiovascular system.

## 5. Conclusions

Patients with JOHD seem to have elevated rHR compared to healthy controls. Additionally, the JOHD participants had significantly decreased SBP compared to the healthy controls. These changes seem to be indicative of progressive neurodegeneration and may further advance our understanding of the neurobiology of JOHD.

## Figures and Tables

**Table 1:** Baseline demographics by groups.

	JOHD Group	Controls	*p*-Value
***N* (Visits)**	27 (64)	259 (395)	NA
**Female, % (*n*)**	55.6 (15)	53.5 (138)	0.998
**Age (years), Mean ± SD**	15.89 ± 6.06	12.34 ± 3.76	<0.001
**CAG Repeats, Mean ± SD**	72.19 ± 14.18	20.29 ± 3.91	<0.001
**Parental SES, % (*n*)**			0.356
**1**	0.0 (0)	0.8 (2)	
**2**	48.0 (12)	58.0 (149)	
**3**	40.0 (10)	37.4 (96)	
**4**	8.0 (2)	3.1 (8)	
**5**	4.0 (1)	0.8 (2)	
**Missing**	*N* = 2	*N* = 2	
**BP Increasing Meds, % (*n*)**	11.1 (3)	2.7 (7)	0.087
**BP Decreasing Meds, % (*n*)**	14.8 (4)	1.2 (3)	<0.001
**Disease Duration (years),**	3.38 ± 3.04	NA	NA
**Mean ± SD**			

BP, blood pressure; CAG, cytosine-adenine-guanine; JOHD, juvenile-onset Huntington’s Disease; SD, standard deviation; SES, socioeconomic status.

**Table 2:** Primary outcomes.

	JOHD Group	Controls	*p*-Value
**rHR, Mean ± SE**	88.56 ± 2.36	78.50 ± 0.83	<0.0001
**SBP, Mean ± SE**	109.96 ± 1.99	115.99 ± 0.71	0.0053
**DBP, Mean ± SE**	61.88 ± 1.17	64.25 ± 0.42	0.060

DBP, diastolic blood pressure; JOHD, juvenile-onset Huntington’s Disease; rHR, resting heart rate; SBP, systolic blood pressure; SE, standard error.
